# Occupational Medicine and Environmental Health in the Border Areas of Euro-Arctic Barents Region: A Review of 30-Year Russian–Norwegian Research Collaboration Outcomes

**DOI:** 10.3390/ijerph17113879

**Published:** 2020-05-30

**Authors:** Valery P. Chashchin, Sergei Gorbanev, Yngvar Thomassen, Evert Nieboer, Dag G. Ellingsen, Sergei Syurin, Alexandr Nikanov, Max Chashchin, Jon Øyvind Odland

**Affiliations:** 1 Northwest Public Health Research Center, 191031 St. Petersburg, Russia; valerych05@mail.ru (V.P.C.); info@znc.ru (S.G.); kola.reslab@mail.ru (S.S.); krl_s-znc@mail.ru (A.N.); max.chashchin@gmail.com (M.C.); 2 National Institute of Occupational Health, NO-0030 Oslo, Norway; yngvar.thomassen@stami.no (Y.T.); dag.ellingsen@stami.no (D.G.E.); 3 Department of Biochemistry and Biomedical Sciences, McMaster University, Hamilton, ON L8S 4K1, Canada; nieboere@mcmaster.ca; 4 Institute of Community Medicine, University of Tromsø, N-9037 Tromsø, Norway; 5 International Research Laboratory for Reproductive Ecotoxicology (IL RET), The National Research University Higher School of Economics, 101000 Moscow, Russia

**Keywords:** occupational health, Russia–Norway collaboration, scientifically based interventions

## Abstract

This year marks 30 years of close collaboration between a consortium of institutions, namely, the Northwest Public Health Research Center, Saint-Petersburg (NWPHRC); the Institute of Community Medicine (ICM) of UiT (The Arctic University of Norway, Tromsø); the National Institute of Occupational Health (NIOH), Oslo; the University Hospital of Northern Norway (UNN) at Tromsø; and McMaster University (MU), Hamilton, ON, Canada. During the early years of cooperation, Dr. Chashchin was the Director of the Scientific Laboratory of the North-West Public Health Centre Branch of the NWPHRC located in the town of Kirovks in the Murmansk Region. The primary focus of this long-standing collaboration was to assess and address issues that are important for maintaining the health of the population living in the border areas of Russia and Norway and included the reduction of occupational health risks among workers employed in mining, metallurgical and machine-building enterprises located in the north-western region of Norway and the adjoining Kola Peninsula in Russia. These industrial activities constituted essential components of the local industries. The ongoing Russian–Norwegian cooperation in the field of occupational medicine is an excellent example of the effective combination of intellectual potential and research technologies of multiple countries. It has resulted in the development of a scientifically based set of measures for practical implementation, contributing to the improvement of working conditions and preservation of the health of workers employed at enterprises where the joint research was carried out.

## 1. Introduction

### History

This year marks 30 years of close collaboration between a consortium of institutions, namely, the Northwest Public Health Research Center, Saint-Petersburg (NWPHRC); the Institute of Community Medicine (ICM) of UiT, the Arctic University of Norway, Tromsø; the National Institute of Occupational Health (NIOH), Oslo; the University Hospital of Northern Norway (UNN) at Tromsø; and McMaster University (MU), Hamilton, ON, Canada. The primary focus of this long-standing collaboration was to assess and address issues that are important for maintaining the health of the population living in the border areas of Russia and Norway and included the reduction of occupational health risks among workers employed in mining, metallurgical and machine-building enterprises located in the north-western region of Norway and the adjoining Kola Peninsula in Russia. These industrial activities constituted essential components of the local industries.

Since 1974, air pollution along the border between Norway and Russia has been recorded in Sør-Varanger County of Norway and, from 1985 on, in Murmansk County, Russia. In May of 1988, the International Symposium on Trace Elements Excess and Deficiency in Arctic/Subarctic Regions took place in Tromsø/Kirkenes, Norway, and served to bring together some of the senior authors.

This led to research cooperation between Dr. Tor Norseth of STAMI (The National Institute of Occupational Health of Norway) and Dr. Valery Chashchin, then of the Kola Institute of Hygiene and Occupational Diseases, Kirovsk [1,2]. Related research papers were presented at the Fifth International Conference on Nickel Biochemistry, Toxicology, and Ecology Issues held in Sudbury, Ontario, Canada, 7–11 September 1992.

The Joint Norwegian-Russian Commission on Environmental Co-operation was established in 1988 and its first meeting was held in 1991 at the ICM. It decided to set up a Health Group, and its report was made public in 1997 [3]. It summarized the findings of a health survey conducted from May to October 1994 among residents living on the Norwegian side of the border with Russia and a comparison group from the Norwegian city of Tromsø. In Russia, the study subjects were selected from the towns of Nikel and Zapolyarny, and the control group was selected from Apatity, Kirovsk, and Umba in the Kola Peninsula. The personal average daily exposure of school children and adults to major atmospheric pollutants was also estimated. This involved questionnaire studies and clinical examinations, as well as the collection of blood and urine samples for analyses. This constituted the first time that these human biomonitoring dimensions were included in a Russian scientific/public health investigation. The report documents the extensive air and water pollution for the border zone due to the industrial emissions/discharges of sulfur dioxide and particulates that contained nickel, copper, and other elements. Significant damage to the vulnerable Arctic environment was clearly evident, and thus, genuine human health concerns for those living in the immediate pollution zones remained. Furthermore, the employees of the mining and metallurgical facilities were likely experiencing even higher health risks [2]. For many years, the Murmansk region was known to have one of the highest occupational morbidity levels in Russia. Nevertheless, the overall conclusion of the Norwegian-Russian Health Study stated, “Our study reveals no major health effects which can be ascribed the nickel and sulphur dioxide pollution on either side of the border” [3].

The activities described above took place throughout the political upheaval and economic crisis after the collapse of the Soviet Union in 1991 and the formation of new countries. The described Norwegian/Russian partnership contributed to the preservation and strengthening of the scientific potential of the NWPHRC and other medical institutions in Northwest Russia. This effort was led by Eiliv Lund of the ICM and Valeri Chashchin and thus took place during the Russian perestrojka (openness) period of the early 1990s. The background for the above investigation was the Norwegian public concern of potential trans-boundary transfer of the pollutants emitted by the local nickel mining/refining industries and their impact on the health of both Russian and Norwegian border residents [1,2,4]. Specifically, fears were voiced about associations with respiratory diseases [5], allergies [6,7], and pregnancy complications, including congenital malformations in newborns [2].

The project “Intergroup Study of Population and Health in Border Regions” was launched in 1994 by UiT and NIOH, and the fieldwork was conducted during the 1994–1995 period; its focus was on residents of the Sør-Varanger region and the Nikel community. In addition to personal medical examinations and quantitative exposure assessments, nickel concentrations in urine were also measured [8]. Adult residents of Apatity and Umba towns in Russia (Kola Peninsula) outside of the heavily polluted areas on the Russian side and those in the city of Tromsø in Norway were used as control sites. The Russian participants were shown to have higher urine nickel concentrations than the Norwegian subjects, regardless of the place of residence. Significant health status issues potentially linked to environmental nickel and sulfur dioxide pollution were revealed among both groups. However, these studies did not provide sufficient evidence of any causal relationship between the effects of trans-boundary transfer of atmospheric pollution and incidences of respiratory or common non-communicable diseases. A potential “protective effect” of nickel allergy (i.e., induced immunologic tolerance) was hypothesized to be the result of ongoing low-level nickel exposure and allergic diseases in children [7]. In the context of protecting the natural environment, the benign health-related findings did not affect recommendations to reduce atmospheric pollution or the carrying out of extensive alterations of the nickel refinery operations to reduce emissions into the atmosphere and nearby water bodies.

In 1996, the pilot project “Reproductive health related to occupational nickel exposure on the Kola Peninsula of Russia” was initiated by the consortium. Its objective was to assess the technical feasibility of a detailed epidemiological study of fertility, spontaneous abortions, congenital malformations defects, and diseases in children. As a result of this project, evidence of specific nickel effects on the reproductive health of exposed subjects was obtained [8]. To address these concerns, the Kola Birth Registry was set up for the towns of Nikel, Zapolyarniy, and Monchegorsk during the calendar years 1996 and 1997. As of May 2002, a total of 22,155 newborns between 1973–1997 had been documented [9]. All diagnoses were registered using the International Classification of Disease, namely, namely the ICD-10 system. Subsequently, the Murmansk County Birth Registry was established [10,11], which includes all births (*N* = 52,253) for the years 2006–2011. Although the registry was discontinued in 2012 for economic reasons, it has provided data for a number of Master and Ph.D. theses. Nevertheless, it has and continues to be a valuable tool for birth outcome studies in the context of improving pregnancy care and pregnancy outcome, as well as of child health.

A characterization of the exposure experienced by workers at the nickel refinery at Monchegorsk in the Kola Peninsula was published in 1999 [12]. Four fractions were measured, specifically, water-soluble, sulfidic, metallic, and oxidic fractions. The survey confirmed relatively high exposures. A comparable assessment of the copper refinery at the same Monchegorsk metal refining complex was conducted some years later [13]. The chemical composition of individual aerosol particles collected in the nickel refinery was examined by electron-probe analysis [14] and transmission and scanning electron microscopy [15] (also see [16]). These detailed analyses were pertinent for insight about the adverse health experienced by the workers (e.g., cancer). Measurements of particulates involved collecting the total aerosols taken in through the nose and mouth, specifically the “inhalable fraction”, specifically those reaching the bronchoalveolar region—the thoracic fraction—as well as the portion penetrating the alveolar region [17].

In 2001–2003, the project “Reproductive health related to occupational nickel exposure on the Kola Peninsula in Russia” was carried out and involved researchers from the ICM, NIOH, McMaster University, as well as the National Institute of Occupational Safety and Health/Centers for Disease Control and Prevention (USA). The project covered four major areas of research: (1) study of personal breathing zone air samples and daily urine samples from nickel plant workers of various jobs; (2) development of the Monchegorsk City birth register in the form of an electronic database covering all childbirth cases and their outcomes for the period from March 1973 to 31 December 2005 in Monchegorsk (26,848 fetuses and newborns); (3) a cohort study of pregnancies in Monchegorsk city using a questionnaire on spontaneous abortion cases among female employees of the “Kola mining and metallurgical company” and other enterprises in Monchegorsk and Apatity; and (4) a reproductive health study of male nickel plant workers in the Kola Peninsula that involved the collection and analyses of semen, urine, and blood serum samples. These studies resulted in new evidence about potentially harmful factors that nickel production had on female and male [8,9,18,19].

In a 2001 study of health disturbances among shipyard workers engaged in manual and semi-automatic electric-arc welding was initiated. The “Health Study of Shipyard Welders Exposed to Manganese” study was conducted in collaboration with NIOH during the 2001–2002 period and had a 5-year follow-up. It involved an examination of neurobehavioral indicators and the collection of blood sera for the analyses of prolactin and other sex hormone concentrations, as well as biomarkers of exposure to manganese and iron aerosols. The second project among these workers dealt with a study of related nervous system (2007–2010) outcomes. Its primary aim was to identify manganese compound exposures, which potentially could induce nervous function disturbances. The third project, namely, “The study of biomarkers of inflammation and coagulation in welders” (2010–2011), again considered the health effects of welding aerosols in terms of the occurrence, severity, and clinical course of systemic inflammation, pulmonary tissue disturbances, and thrombosis. For the first time in Russia, these studies made it possible to formulate guidance about biomarkers of exposure, related toxic effects, and individual susceptibility, as well as issues related to assessing and managing individual risks of exposure to ultrafine welding aerosol fractions. The findings again illustrated the importance and role of effective measures in reducing occupational exposure and related health problems [20,21,22,23,24].

In 2007–2009, the study “Health of Russian Miners Exposed to Whole-body Vibration” was conducted in a joint effort between STAMI and UiT. It included measurements of whole-body and local vibration parameters experienced by underground mineworkers at the Apatite Joint-Stock Company. It involved clinical examination and the completion of a questionnaire. This joint effort resulted in the development and implementation of effective preventive measures, which improved working conditions and health. Specifically, these efforts contributed to a significant reduction (more than 2-fold) of the incidence of occupational polyneuropathy of the upper extremities and eliminated disorder cases caused by whole-body vibration. These joint efforts helped to harmonize the Russian–Norwegian approaches to the assessment of industrial vibration and clinical protocols pertinent to vibration pathology [25].

During 2012–2014, Finnish and Swedish experts in occupational medicine from Oulu (Finland) and Umeå (Sweden) Universities joined the Russian–Norwegian mine studies. The objective of this collaborative study, titled “Mine Health Sustainability of Miners’ Well-being, Health and Work Ability in the Barents Region—a Common Challenge,” was intended to provide long-term health stability in the Barents region for mining workers exposed to cold, whole-body, and local vibration, as well as to dust and fine particle aerosols of diesel exhaust. Based on an examination of markers of inflammation and coagulation, new scientific data were obtained that confirmed increased risks of circulatory and respiratory diseases related to the inhalation of diesel engine exhaust aerosols generated by mining equipment operated at low air temperatures. The new funding was used to generate recommendations for employers and healthcare professionals to maintain and enhance the health of mineworkers engaged in facilities located in cold climates.

Concurrent with the mining project, the “Food and Health Security in the Norwegian, Finnish, and Russian Border Region Linking Industries, Communities and Socio-economic Impacts” project was initiated. It included questionnaire studies among the inhabitants of the towns of Nikel and Zapolyarny in the Murmansk region, as well as in the border regions of Norway and Finland. It involved sampling blood from Nikel and Zapolyarny residents and puerperas in the Pechenga district of the Murmansk Region; sampling of local food products (fish, poultry, mammals, mushrooms, berries); and chemical analysis of food, water, and blood samples to assess pollutant concentrations. Sanitary and epidemiological data on the food, water contamination, and health of Pechenga district populations were also collected. The study revealed increased levels of average daily intake of metals in the body and non-carcinogenic and carcinogenic risks to the health of the Pechenga district populations exposed to toxic metals contained in local food and drinking water. These findings led to the development of recommendations for reducing and eliminating the consumption of certain local food products and measures for removing nickel from drinking water.

In order to strengthen cross-border cooperation in solving common hygiene problems, the Russian–Norwegian network “Occupational Hygiene in the North—Acquiring and Exchanging Cross-border knowledge” was initiated in 2017–2018 by the ICM and the Department of Occupational and Environmental Medicine of The University Hospital of Northern Norway (UNN), Tromsø. The topics of the three workshops were the issues of choosing and using personal protection equipment in the Far North, assessment of similarities and differences in occupational health in Norway and Russia, and comparison of standards and legislation of Russia and Norway in the field of labor protection.

## 2. Current Activities

Currently, the NWPHRC in cooperation with STAMI is participating in two ongoing Norwegian–Russian research projects:

(a) “Development of methodology for monitoring assessment, predicting and prevention of risks related to transfer of toxic pollutants through biological pathways capable of accumulating in trophic chains and spreading in the Arctic ecosystems” in cooperation with the Northern (Arctic) Federal University named after M.V. Lomonosov Arkhangelsk (NArFU);
(b) “Assessment of Occupational Exposure to diesel exhaust gases among primary aluminium production workers”.

As a result of these studies, the guidelines “Procedure for the development and implementation of measures to reduce morbidity and mortality of population exposed to harmful effects of persistent eco-toxicants in the Arctic zone of Russian Federation” were prepared and claimed for invention. The report “Means for increasing body resistance in post-hypothermic period” was completed.

In general, cooperation with Norwegian colleagues has allowed researchers of the North-West Public Health Research Center to reach a higher operating level in resolving occupational hygiene and occupational medicine issues. Among the breakthrough directions from the collaboration, the following activities are highlighted:

(a) Use of individual exposure to a harmful occupational factor, with subsequent determinations of personal risks of ill-health development. This enhanced the battery of methods available for use in group risk assessment research findings on harmful factor levels at workplace areas;
(b) Determination of a set of biomarkers for the early detection of pulmonary tissue damage, biomarkers of inflammation and coagulation, and biomarkers of damage when exposed to welding aerosols;
(c) Improvement of female and male reproductive health assessment with compulsory consideration of adverse occupational factor and cold effects on the reproductive function of industrial enterprise workers; development of a birth registry in the Murmansk Region, including data on expectant mothers exposed to harmful occupational factors;
(d) Study of whole-body and local vibration characteristics generated by modern mining equipment and its effect on the health of above and underground mine workers;
(e) Assessment of the effect of ultrafine particle aerosols from diesel exhaust gases on cardiorespiratory system health of mining enterprise workers;
(f) Assessment and regulation of cold factors among workers engaged in open areas using a set of specific biomarkers.

The major results of the Russian–Norwegian collaboration studies have been the acquisition of scientific knowledge and its practical implementation (along with other measures), permitting significant improvements in working conditions and a reduction in occupational morbidity in the mining and metallurgical industry in the Murmansk region. A number of occupational diseases with severe health outcomes among nickel production workers have not been sufficiently reported, specifically, toxic myocardiopathy, toxic hepatitis, and toxic pneumosclerosis, which are predominantly caused by nickel tetracarbonyl.

In addition to scientific achievements, it is important to evaluate the social benefits of the Russian–Norwegian cooperation in the field of occupational medicine, for example, the assessment of indicators that characterize changes in the intensity of harmful occupational outcomes, as well as the elimination of specific occupational diseases at enterprises where research and practical measures have been implemented, namely, nickel-induced contact dermatitis, cobalt-related cardiomyopathy, whole-body vibration disease, and pneumoconiosis. The incidence of other previously prevalent occupational diseases has been significantly reduced. The number of newly diagnosed cases of such diseases among workers employed in the metallurgical industry in the Kola region decreased from 126 to 32 cases per year among 10,000 employees during the 2013–2017 period, while the incidence rate among workers in the entire Murmansk region decreased from 10.6–6.6 per 10,000 employees over the same period.

Most occupationally exposed patients lived in Kirovsk and Apatity (622 persons), while in the vicinity of the largest mining and metallurgical enterprises in the nearby Pechenga district and town of Monchegorsk, there were 411 and 538, respectively. The maximum decrease in the number of work-related disease patients for the period 2007–2017 was observed in the Pechenga district (from 82–14 persons, or 5.86 times) and Monchegorsk (from 48–15 persons, or 3.20 times), where the enterprises for the extraction and processing of copper–nickel ore are situated. The report concluded that the risk of developing occupational diseases sharply decreased in the Pechenga region (Gorbanev S., 2019 Annual report of the NWPHRC).

A list of the various reports and documents generated by the research activities described are provided in the Appendix A, Appendix B and Appendix C. 

## 3. Conclusions

The ongoing Russian–Norwegian cooperation in the field of occupational medicine is an excellent example of the effective combination of the intellectual potential and research technologies of multiple countries. It has resulted in the development of a scientifically based set of measures for practical implementation, contributing to the improvement of working conditions and preservation of the health of workers employed at enterprises where the joint research was carried out.

## Appendix A. Reports of the Main Studies (Not Peer Reviewed)

- Reproductive and Developmental Health in Relation to Occupational Exposure to Nickel in the Kola Peninsula of Russia: A Feasibility Study (28 April 1997), Final Report, 133p.
- Reproductive and Developmental Health in Relation to Occupational Exposure to Nickel in the Kola Peninsula of Russia: A Feasibility Study (Revised 24 July 1997), Final Report, 149p.
- The Norwegian—Russian Health Study 1994/95. A Cross-sectional study of pollution and health in the border area. University of Tromso (Norway), 1997, 124p.
- Environmental and occupational exposure, life-style factors and pregnancy outcome in Arctic and subarctic populations of Norway and Russia. Institute of Community Medicine, UiT the Arctic University of Norway, Tromsø, 2000, 160p. Odland Ph.D. Thesis.
- Report. Assessment of exposure to whole body vibration at the Apatiti OAO, Kirovsk mine. Tromso, 2007, 31p.
- Report. Assessment of exposure to whole body vibration at the OAO Apatity, open mine Kirovsk Kola Peninsula. Tromsø, 2008, 47p.
- Mine Health 2012–2014: Sustainability of miners’ wellbeing, health and work ability in the Barents region—A common challenge. Guidebook on cold, vibration, airborne exposures and socioeconomic influences in open pit mining.

## Appendix B. Published Articles and Documents

- Approximately 85 publications in scientific Russian and international journals, as well as in presentations at international conferences (in Russia, Norway Canada, Finland, South Africa, and the USA.
- Eighteen procedural documents (guidelines, manuals for physicians, infomethodic letters) in Russia.
- Three regulatory documents (sanitary rules, temporary sanitary and hygienic requirements, safety rules) in Russia.
- Four Ph.D. and three candidate theses defended by employees of the North-West Public Health Research Center.

## Appendix C. Ongoing Cooperation

Ongoing concerns and the results of the Russian-Norwegian-Canadian studies desribed in this article, as well as those of the five-sided cooperation between scientists from Russia, Norway, Canada, Finland and Sweden, were discussed and reviewed at four symposia during the 2008–2014 period. These took place in Kirovsk (Russia), Tromsø (Norway), Oulu (Finland) or Umeå (Sweden) under the general title “Ecology and Health Protection of Industrial Workers in the Barents”.
